# Spatial Differentiation and Influencing Factors in the Ecological Well-Being Performance of Urban Agglomerations in the Middle Reaches of the Yangtze River: A Hierarchical Perspective

**DOI:** 10.3390/ijerph191912867

**Published:** 2022-10-08

**Authors:** Yuanyuan Zhu, Rui Zhang, Jiaxing Cui

**Affiliations:** 1 Key Laboratory for Geographical Process Analysis and Simulation of Hubei Province, Central China Normal University, Wuhan 430079, China; 2 College of Urban and Environmental Sciences, Central China Normal University, Wuhan 430079, China

**Keywords:** sustainable development, ecological well-being performance, hierarchical effect, resource and environmental consumption, human–environment interactions, threshold regression model

## Abstract

Improving the ecological well-being performance (EWP) of natural resources and environmental consumption in relation to human well-being, within the ecological boundary, is necessary for sustainable development. This study used the Super-SBM model to measure the urban EWP of urban agglomeration in the middle reaches of the Yangtze River (MRYRUA) in 2020. The spatial differentiation characteristics of EWP in the MRYRUA were identified. The heterogeneity in the direction and size of the influencing factors of EWP at different urban hierarchy (UH) levels was empirically tested by establishing a threshold model. The results are as follows: (1) In 2020, the EWP of the study area showed a trend of high levels in the southwest and low levels in the northeast. The EWP presented a multi-center “core–periphery” distribution, and the characteristic of “central collapse” was evident. The UH level of the middle and lower hierarchy-level cities was inconsistent with its EWP. (2) A non-single linear relationship was found between the influencing factors of the EWP of the MRYRUA and the EWP. The impacts of technological progress, industrial structure, environmental regulation, and population density on the EWP of the MRYRUA all showed threshold characteristics. (3) Heterogeneity and stages were both observed for the influencing factors of EWP under different UH levels. The effect of technological progress on EWP presented the characteristics of bidirectional and two-stage developments, and environmental regulation presented the features of a significant positive three-stage development. Both industrial structure and population density presented two-stage aspects, but the former acted in a negative direction, while the latter served in a positive order. This study provides a theoretical basis for the government to formulate differentiated regional policies and promote the coordinated improvement of EWP among cities at all hierarchy levels in the urban agglomeration. This study is of great significance to the sustainable development of urban agglomerations. Its results can provide a reference for other urban agglomerations, metropolitan areas, and city clusters worldwide to coordinate economic development, ecological protection, and to improve people’s well-being.

## 1. Introduction

The world is facing threats such as resource shortages, environmental pollution, ecological imbalance, climate change, and global poverty. How to coordinate the relationship between resource and environmental consumption, economic growth, and improvement of well-being within the ecological boundary has become a hotspot of global concern in recent years [1]. According to the United Nations Department of Economic and Social Affairs [2], urbanization will continue in more developed and less developed regions, with urban areas hosting 68% of the world’s population by 2050. Although global urbanization promotes economic prosperity and social progress, it has also brought severe ecological and environmental problems [3], such as ecological deterioration, environmental pollution, and excessive resource consumption, causing increasingly severe coercive effects and also far-reaching effects in regard to resources and the environment. This situation is challenging in urban agglomerations with high population density, as these factors restrict the improvement of human well-being and are unconducive to sustainable development [4]. In this context, the solution of how to coordinate the relationship between the economy, society, and the environment and improve people’s well-being has become the focus of governments, related organizations, and researchers. The United Nations 2030 Sustainable Development Goals (SDGs) propose to promote human well-being in poverty reduction, education, health, and social equity, as well as solve environmental problems in climate change, resource utilization, and terrestrial ecology [5]. These goals therefore aim to promote the sustainable development of the economy, society, the environment, and to improve human well-being [6]. Based on the SDGs, the United Nations Sustainable Development Solutions Network conducted a dynamic assessment on the sustainable development goals of European and American cities. Even the top cities in the index were still far from achieving sustainable development goals [7]. The 2022 Sustainability Report also showed that under the confluence of multiple international crises—such as COVID-19, climate change and the rise of regional conflicts—the progress of SDGs, which is a global goal, has stalled [8]. In its America 2050 strategic spatial plan, the United States formulated corresponding plans to deal with issues such as the energy crisis and global climate change, inefficient land resource use, and ecosystem pressure in mega metropolitan areas [9]. China has proposed a series of major national strategies, such as ecological civilization construction and high-quality development. The government of China wishes to further coordinate the relationship between economic development, ecological environment, and people’s well-being and, as a result, accelerate the transition from “economic well-being” to “ecological well-being”. These developments show that balancing the relationship between economic growth, ecological environment, and the improvement of well-being still requires further exploration. Sustainable development requires minimizing the impact on resources and the environment and maximizing human well-being [10,11,12], indicating a greater emphasis on improving ecological well-being performance (EWP). With the strengthening of mobility of economic resources, and other factors of production in regional urban networks, scattered cities have gradually evolved into highly integrated clusters of cities and urban agglomeration [13]. Urban hierarchy (UH) is based on the status and role of all cities in the urban system, and it also determines the scale and function type of the towns in the urban system to some extent [14]. Cities of different sizes in the urban agglomeration have formed a hierarchical structure with orderly scales and reasonable division of labor, which is an essential cornerstone for improving collective efficiency [15]. Improving the overall EWP of urban agglomerations, based on giving full play to the role of cities at all hierarchical levels and achieving sustainable development, is a practical problem facing China and other countries. Therefore, it is necessary to study the spatial differentiation of EWP according to the hierarchical characteristics of cities in urban agglomerations. It is also necessary to analyze the differences in the direction and extent of the influence that factor in EWP, against the backdrop of different hierarchy levels. In this way, different reference paths can be provided for cities at different levels to improve EWP.

EWP refers to the efficiency of converting natural resources and environmental consumption into human well-being [16,17]. It comprehensively considers economic, environmental, and social factors and reflects the degree of coordination between human well-being improvement and ecological resource consumption [18]. Daly [19] was the first to put forward the concept of EWP, which evaluates the level of regional sustainable development by the well-being that is improved via a unit of natural consumption. However, Daly did not define specific quantitative indicators of natural resource consumption, so the EWP concept was only a prototype. Only when Rees proposed the ecological footprint (EF) theory [20] in 1992 did the measurement research of EWP become popular. Subsequently, the concept of EWP, in considering human well-being and environmental impact, has been proposed successively, such as in the Happy Planet Index [21] and Environmental Efficiency of Well-Being (EWEB) [22]. They are all expressed by the ratio of human well-being indicators (such as life expectancy and happiness satisfaction) to ecological resource consumption indicators (such as EF and energy consumption). Zhu et al. [23] defined EWP as the efficiency of converting natural consumption into human well-being. They expressed it as the Human Development Index (HDI) ratio to EF. Methods to measure EWP mainly include the ratio [24,25] and model methods. Compared with the former, the latter has been widely used in research in recent years. At present, the widely used models mainly include data envelopment analysis (DEA) [16,26], stochastic frontier analysis (SFA) [27], and the regression equations method [22].

In recent years, the research on EWP mainly focused on three aspects. The first aspect is the empirical measurement of EWP at the national, regional, and city levels. Dietz et al. [28] used panel data from 58 countries, from 1961 to 2003, and found that per capita GDP had a U-shaped relationship with the environmental intensity of human well-being. Knight et al. [25] assessed the degree of change in the relationship between the per capita EF and life expectancy in 116 countries. They found that decoupling between environmental consumption and human well-being, and the degree of decoupling in developed countries was more significant than in less developed countries. Zhang et al. [29] found that most developed and G20 countries generally had low eco-efficiency in improving human well-being. Li et al. [30] found that China’s inter-provincial EWP generally showed a downward trend with apparent differences between provinces. Long et al. [16] calculated the EWP of 35 large cities in China in 2013 and found that the EWP of these cities was not high, and the gap between cities was large. Wang et al. [31] measured the EWP in the Poyang Lake area at the local level. These studies all manifestly show that economic development is inconsistent with the EWP, and EWP presents different characteristics in different countries and cities.

The second aspect is research on the regional and spatial differences in EWP. Fang and Xiao [32] found that China’s inter-provincial EWP generally showed a distribution pattern of “eastern > western > central” and showed a strong spatial correlation. The empirical analysis of 278 Chinese cities by Bian et al. [33] instead found that the EWP of Chinese cities presented a distribution pattern of “central > eastern > western.” Further, Wang et al. [34] found that the EWP in the middle and lower reaches of the Yangtze River Economic Belt presented a significant “center–periphery” spatial structure. Xia and Li [35] measured the EWP of the Beijing–Tianjin–Hebei urban agglomeration from 2006 to 2019. They found that its EWP showed a “polycentric” network pattern and a radial spatial structure over time. As such, these studies all indicate regional imbalances and spatial heterogeneity in EWP.

The third aspect is research on the factors influencing EWP. Koziuk et al. [36] found that educational and scientific investment in less developed countries had a more significant impact on EWP than in developed countries. Knight et al. [22] found that economic development had a negative quadratic effect on EWEB, income inequality also had a negative effect, and social capital had a positive effect. Deng et al. [37] found that economic growth, degree of openness, environmental regulation, industrial structure, and technological level all significantly impacted EWP’s improvement. However, the direction and intensity of the effects were different in different regions. Long et al. [16] found that urban population density significantly and positively affected urban EWP. Wang et al. [18] found that the degree of openness and industrial structure had a heterogeneous impact on EWP over time. Wu et al. [38] assessed the impact of green transition on EWP. Bian et al. [39] found that there was a “U-shaped” relationship existing between urbanization level and the EWP. Hu et al. [40] found that government macro-control had inhibitory effects, whereas consumption levels, industrial structure upgrades, and technological innovation had catalyzed effects. Therefore, factors such as industrial structure, technological progress, environmental regulation, population density, urbanization level, local government influence, and degree of openness have an essential impact on EWP. However, the direction and size of the influencing factors show differences for regions that are of different natures, scales, and types. This non-linear relationship should be discussed further.

Although researchers have conducted many explorations around EWP, some areas remain unexplored. (1) Existing studies were often carried out on a large scale, such as at the national, regional, and provincial levels. However, the analysis based on the urban agglomeration level is relatively lacking. Larger spatial scales tend to make researchers ignore the spatial heterogeneity of smaller regions [41]. Urban agglomerations are highly populated areas and critical spatial units for improving the living environment [42] and studying their urban EWP. (2) Human well-being covers economic, social, environmental, and other aspects [43]. Previous studies mostly used the HDI as an indicator to measure human well-being, ignoring the direct well-being brought by an excellent ecological environment. A good ecological environment such as an urban green space has positive effects such as relieving fatigue, pleasing the mind and body, promoting physical and mental health, promoting the city’s livability, and improving residents’ well-being [44,45]. Therefore, favorable environmental well-being needs to be considered. (3) Most studies failed to explore EWP’s influencing factors in different cities deeply enough; as such, they could not identify differentiated improvement paths. Different path measures must be taken according to local conditions in order to improve the efficiency of urban agglomerations [46]. Heterogeneity is found in the effect of EWP’s influencing factors, especially in cities of different hierarchy levels. The single linear regression method cannot adequately reflect the structural, non-single linear relationship between the influencing factors and EWP.

The urban agglomeration in the middle reaches of the Yangtze River (MRYRUA) has played an essential role in the green development process of the Yangtze River Economic Belt [47]. However, the rapid development of industrialization and urbanization has led to many ecological and environmental problems; for example, it has led to restricting the improvement of people’s well-being, and the hindering of sustainable development [48]. Therefore, the MRYRUA, during 2020 as the research period, was selected as the case site for the study of EWP. From the perspective of urban hierarchy, the spatial differentiation characteristics of EWP of the MRYRUA were identified. The threshold regression model was used to analyze the hierarchical differences and stages of the influencing factors of EWP. The contribution of this study is in the construction of a comprehensive EWP evaluation index system, which covers economic, social, and environmental dimensions; further, the relationship between EWP and urban hierarchy was explored. Moreover, according to the urban hierarchy of urban agglomeration, a non-linear regression model was used to identify the hierarchical heterogeneity of the influencing factors of EWP. This is important, as it is not addressed by existing studies. This study aims to help the government to tailor policies to local conditions and provide a reference for promoting the high-quality development of urban agglomerations and therefore achieving sustainable development.

## 2. Materials and Methods

### 2.1. Study Area

**Figure 1:**
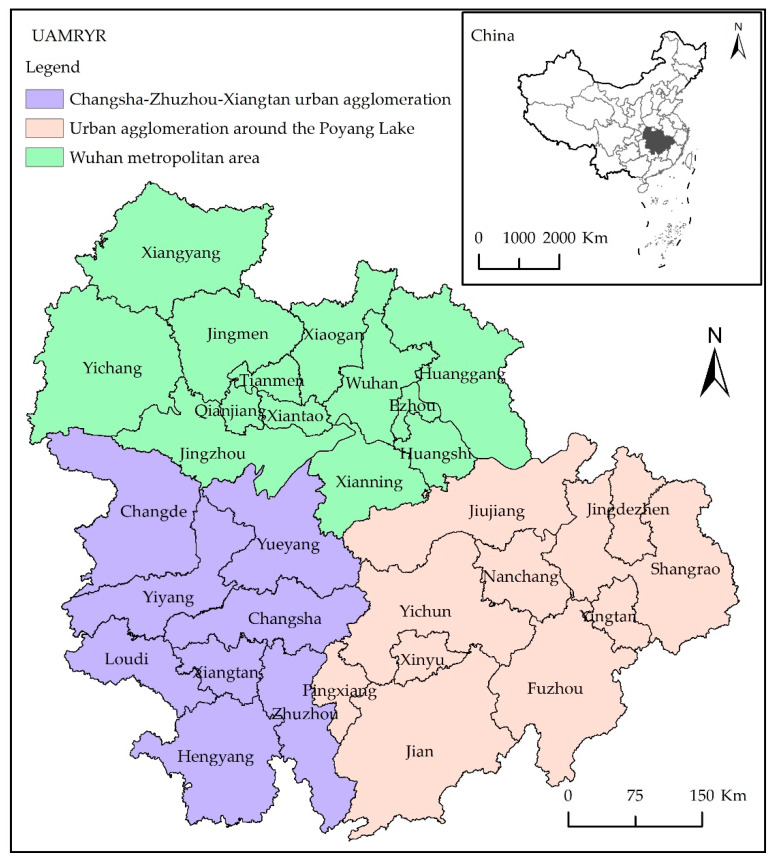
The location of the study area.

The MRYRUA is located in the central region of China. According to the Development Plan for Urban Agglomeration in the Middle Reaches of the Yangtze River [49], the MRYRUA is a super-large urban agglomeration mainly formed by the Wuhan metropolitan area, the Changsha–Zhuzhou–Xiangtan urban agglomeration, and the urban agglomeration around Poyang Lake (Figure 1), which includes 31 prefecture-level cities. It is the largest urban agglomeration in China, spread over 317,000 square kilometers (3.3% of China’s land) and with a population of 130 million (9.4% of China’s total population) in 2020. The MRYRUA has typical multi-center characteristics and has gradually formed a hierarchical and deep-level urban system [50]. The MRYRUA is a crucial area for implementing the strategy of promoting the rise of central China, thereby promoting new urbanization, and building a beautiful China. It has become a significant growth pole for China’s economic development and a primary space for building an ecological security barrier [51]. However, the development of the MRYRUA is relatively low, and it is still in the stage of rapid development [52]. The pressure on population, resources, and the environment is tremendous, and the problem of environmental pollution is prominent. The coordination between economic development, ecological environment, and people’s well-being needs to be urgently improved [53]. The 14th Five-Year Plan of China [54] has proposed optimizing the internal spatial structure of urban agglomerations; building ecological and security barriers; and forming multi-center, multi-hierarchy level, and multi-node urban network urban agglomerations. Therefore, evaluating the EWP of the MRYRUA from a hierarchical perspective is of great significance to its sustainable economic, social, and environmental development.

### 2.2. Data Source

The evaluation units were 31 prefecture-level cities in the MRYRUA. The space vector data came from the Resource and Environmental Science and Data Center website of the Chinese Academy of Sciences [55]. The socio-economic data were mainly sourced from the China City Statistical Yearbook (2021), China Urban Construction Statistical Yearbook (2020), Hunan Statistical Yearbook (2021), Hubei Statistical Yearbook (2021), and the Jiangxi Statistical Yearbook (2021). Other data were sourced from the statistical yearbooks of relevant cities, the statistical bulletin of national economic and social development, the statistical bulletin of water resources, and the bulletin of environmental statistics. The average years of education were obtained from the Seventh National Census Bulletin of the respective provinces.

### 2.3. Evaluation Indicators

#### 2.3.1. Ecological Well-Being Performance

EWP refers to the efficiency with which natural resources and the ecological environment are transformed into a corresponding level of human well-being. This study referred to relevant studies such as Bian et al. [26], Yao et al. [56], Xia and Li [35], Wang et al. [18], and Fang and Xiao [32]. Based on the input–output theory—with ecological resource consumption and ecological environment destruction as cost-type input factors, and human well-being as benefit-type output factors—an EWP evaluation index system was constructed (Table 1). The goal of inputting natural resources and ecological environmental elements into the analytical method is to promote human development and improve well-being. The resources and ecological environmental elements can be divided into natural resource consumption and ecological environmental destruction. Human well-being refers to the living state of health, happiness, and material wealth, which can be further divided into subjective and objective well-being [57]. Objective well-being indicators include economic growth, social equality, universal education, health, and favorable environment. Further, environmental pollution appears as output in actual production. Nevertheless, according to the connotations of EWP, it can be regarded as a cost. Therefore, wastewater, waste gas, and solid waste discharge characterize ecological environmental damage inputs. In addition, some studies have found that income inequality in China significantly and negatively impacts residents’ subjective well-being [58]. The excessive income gap is not conducive to improving residents’ happiness. Owing to the poor availability of subjective well-being data at the urban scale, this study selected the level of equalization of urban and rural residents’ income to represent subjective well-being indirectly.

#### 2.3.2. Urban Hierarchy

**Figure 2:**
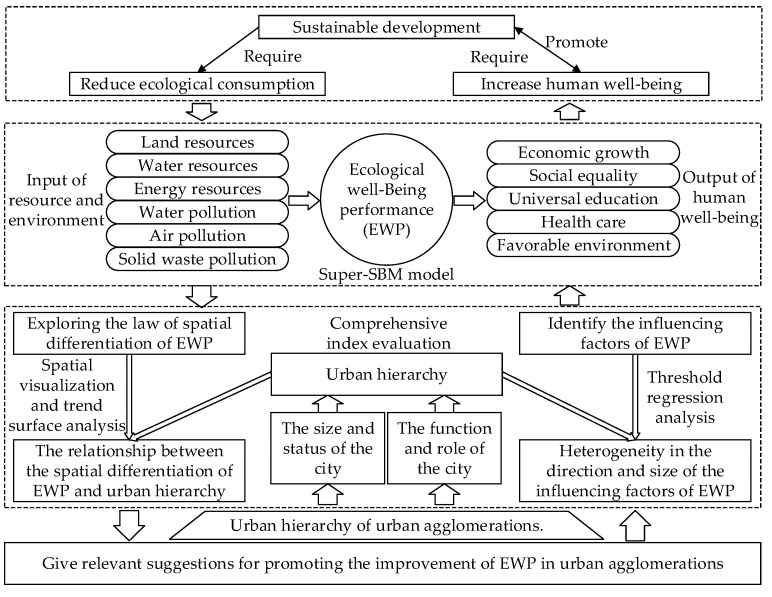
A research framework on spatial differentiation and influencing factors of EWP in urban agglomerations from a hierarchical perspective.

In 1933, central place theory first systematically defined the spatial organization and structure of the urban agglomeration [59]. The theory conducted a theoretical discussion on UH. At present, researchers mainly measure the UH level of cities through three methods. (1) The centrality of a city is measured directly through the city’s index system as a city hierarchy [60,61,62,63]. (2) The centrality of cities is measured with the help of network analysis tools [64,65]. (3) The hierarchical system of cities is explained from the perspective of inter-city connections, such as gravity models and transportation networks [50,66,67]. Centrality refers to the relative importance of a central place in serving its peripheral areas and indicates the level of urban centrality [60]. Centrality can evaluate the city’s status in the urban system, and the urban system hierarchy can be divided by it [68]. It is a spatial composite of hierarchical and network architectures [69]. The transportation network reflects the degree and level of a city’s internal and external connections. It is an important aspect of urban functions, and the hierarchical system it represents reflects the relationship between the city and the distribution of the entire urban system [70]. Overall, the UH not only reflects the comprehensive strength level and status of the city, but also reflects the role that the city plays in the urban system in which it is located. The essence of UH upgrading is to give full play to the city’s leading, driving, and radiating role in the urban system [71,72]. Accordingly, this study built an evaluation index system for the UH level based on the city’s size and scale and the city’s external impact and role (Table 2). The theoretical framework of this study is shown in Figure 2.

### 2.4. Research Method

#### 2.4.1. Super-SBM Model

The Super-SBM model has often been used to measure input–output efficiency. Compared with the traditional DEA model, it can solve the slack problem of input and output variables [74]. It can also solve the problem in which multiple decision-making units (DMUs) are effective simultaneously and cannot be sorted [75]. The above advantages make it widely favored in the study of EWP [18,56]. Therefore, this study used the Super-SBM model to measure the EWP of the MRYRUA. The model can be expressed as follows:

(1) $$\rho *=\frac{1-\frac{1}{M}\sum_{m=1}^{M}\frac{s_{m}{}^{-}}{x_{mk}}}{1+\frac{1}{N}\sum_{n=1}^{N}\frac{s_{n}^{+}}{y_{nk}}}$$

(2) $$s.t.\left\{\begin{array}{l} x_{k}=\sum_{i=1}^{I}\lambda_{i}x_{mi}+s_{m}{}^{-},m=1,2,\cdots ,M\\ y_{k}=\sum_{i=1}^{I}\lambda_{i}y_{ni}-s_{n}{}^{+},n=1,2,\cdots ,N\\ \sum_{i=1}^{I}\lambda_{i}=1,i=1,2,\cdots ,I\\ \lambda_{i}\geq 0,s_{m}{}^{-}\geq 0,s_{n}\geq 0 \end{array}\right.$$

In Formulas (1) and (2), ρ* is the efficiency value of EWP in the MRYRUA; *M* and *N* denote the number of input and output variables, respectively; *I* denotes the number of DMU; *s_m_*^−^, *s_n_*^+^ represent the slack of input and output; *x_m_*, *y_n_* are input and output vectors; *x_mk_*, *y_nk_* are input and output, respectively; λ*_i_* is the weight coefficient; and *i* represents the DMU *i*. When ρ* ≥ 1, it means that the decision-making unit is entirely effective, and both *s_m_*^−^ and *s_n_*^+^ are 0; when ρ* < 1, it means that the efficiency of the DMU is lost.

#### 2.4.2. Comprehensive Index Evaluation Method

This study adopted the comprehensive index evaluation method in order to evaluate the UH level. First, the range standardization method was used to process the original data in order to eliminate the dimensional differences uniformly. Second, the entropy weight method was used to determine each indicator’s weight objectively. Finally, the total value of each weighted index was calculated. The specific calculation formula is found in related research [76].

#### 2.4.3. Trend Surface Analysis

The trend surface approximates the actual surface, which can simulate the spatial distribution law and variation trend of geographic elements [77]. This study used trend surface analysis to explore regional EWP’s overall spatial differentiation trend. Suppose *Z_i_* (*x_i_*, *y_i_*) is the EWP value of the city, and (*x_i_*, *y_i_*) is the plane space coordinate. The trend surface function can be expressed as follows:

(3) $$Z_{i}(x_{i},y_{i})=T_{i}(x_{i},y_{i})+\varepsilon_{i}$$

(4) $$T_{i}(x_{i},y_{i})=\beta_{0}+\beta_{1}x+\beta_{2}y+\beta_{3}x^{2}+\beta_{4}y^{2}+\beta_{5}xy$$

In Formulas (3) and (4): *Zi* (*xi*, *yi*) is the trend function, which represents the trend value in an extensive range. This study used a second-order polynomial to measure the trend value of regional EWP; *Ti* (*xi*, *yi*) is the trend function; and *εi* is the random autocorrelation error, which represents the deviation between the actual value and the trend value of the EWP in the city *i*.

#### 2.4.4. Threshold Regression Model

Threshold regression can mine the jumping or abrupt change rules between variables and is often used to study the heterogeneity of interactions between variables [78]. The threshold regression model proposed by Hansen [79] can divide the data interval endogenously according to the characteristics of the data itself, thereby avoiding the randomness of artificially dividing the sample interval. In the background of different UH levels, a nonlinear relationship exists between various influencing factors and EWP. Traditional linear regression does not explain this relationship well and using a threshold regression model is closer to reality. Therefore, this study adopted Hansen’s threshold regression model and set the following single threshold regression model as follows:

(5) $$Y_{i}=\lambda_{0}+\lambda_{1}D_{i}•I(T_{i}\leq \delta )+\lambda_{2}D_{i}•I(T_{i}>\delta )+\lambda X_{i}+\varepsilon_{i}$$

In Formula (5), *Y_i_* is the explained variable of the city *i*. *I* (•) is the indicative function, which takes the value of 1 when the expression in the brackets is true and the value of 0 when it is false. *D_i_* is the core explanatory variable, and *T_i_* is the threshold variable. *δ* is the threshold value, *X_i_* is the control variable, and *λ* is the coefficient of the control variable *X_i_*; further, *λ*_0_ is the constant term and *ε_i_* is the random disturbance term. When *T_i_* ≤ *δ*, the core explanatory variable *D_i_*’s coefficient is *λ*_1_, and when *T_i_* > *δ*, the core explanatory variable *D_i_*‘s coefficient is *λ*_2_. Formula (5) only assumes one threshold, and the test of two or more thresholds can be deduced.

## 3. Results

### 3.1. Spatial Differentiation Characteristics of EWP

**Figure 3:**
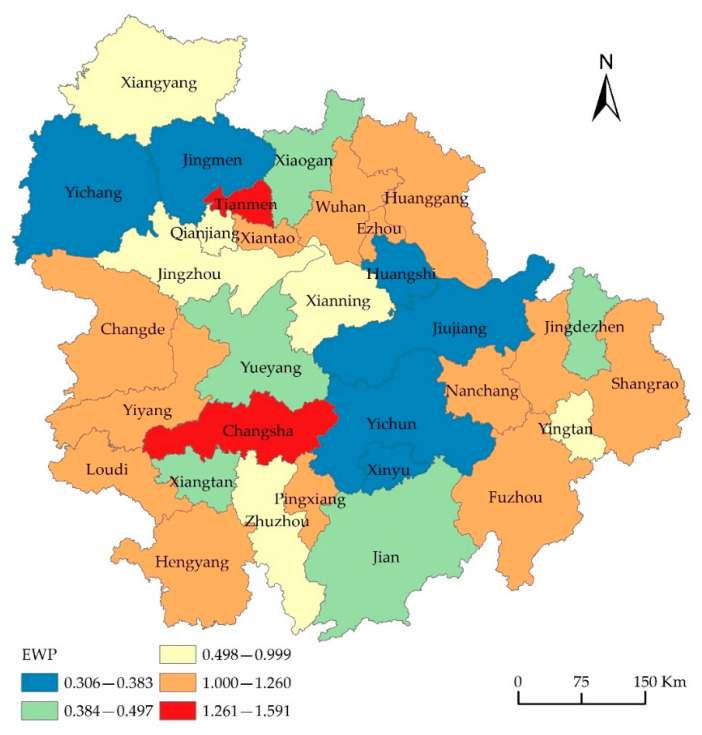
Spatial distribution of EWP in MRYRUA.

**Figure 4:**
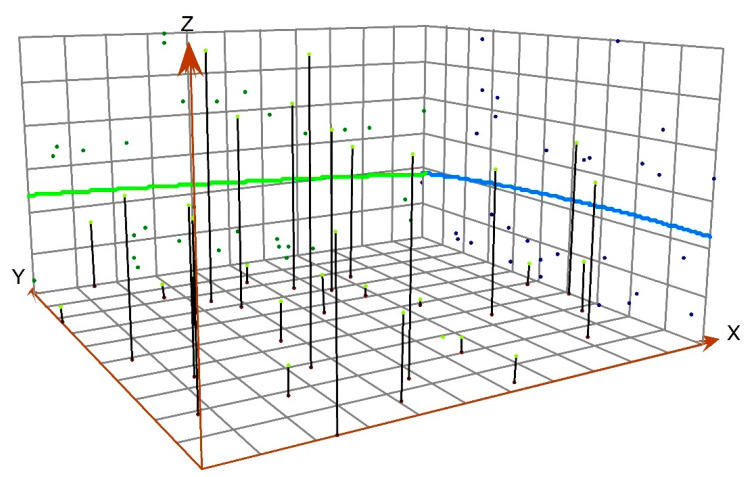
Trend analysis results (the arrow on the X-axis indicates an eastward direction. the arrow on the Y-axis indicates a northward direction. The Z-axis indicates the EWP value. The green line indicates the trend of EWP in the east-west direction. The blue line indicates the upward trend of EWP in the south and north).

EWP was graded using the natural breaking point classification method. The spatial distribution of EWP of the MRYRUA (Figure 3) was visualized through ArcGIS10.2. The trend surface analysis graph (Figure 4) was visualized by the trend analysis tool.

The EWP of the MRYRUA showed a trend of higher performance in the southwest and lower in the northeast. Further, the multi-center “core–edge” distribution trend was remarkable. At the junction of the three sub-urban agglomerations, the characteristic of “central collapse” was prominent. The UH level of the city did not match the level of EWP. Specifically (Figure 2, Table 3), the EWPs of 14 cities, including Changsha, Tianmen, and Wuhan, have reached the DEA adequate frontier level (EWP ≥ 1), which accounts for 45.16% of the total cities in the region. The central cities of the three sub-urban agglomerations and its surrounding cities, have formed high-value clusters of EWP. The “core–periphery” hierarchical structure with Wuhan, Changsha, and Nanchang, as the core, was apparent. The EWPs of 17 cities, including Jiujiang, Huangshi, Yichun, and Xiangyang, failed to reach the adequate frontier level of DEA (EWP < 1), which accounts for 54.84% of the total cities in the region. Cities with low and medium EWPs were mainly distributed at the junction of three sub-urban agglomerations, showing the spatial characteristics of “central collapse.” From a hierarchy perspective, the EWPs of the three urban agglomeration central cities (Wuhan, Changsha, and Nanchang) with high UH levels had reached an effective level. The EWP levels of Changsha and Wuhan were at the forefront of all cities. Some cities in the second tier of the UH level, such as Xiangyang and Yichang, did not show a high value of EWP. However, low UH level cities with higher levels of EWPs were also observed. Examples include Tianmen, Xiantao, and Ezhou, whose performance levels ranked second, fourth, and sixth, respectively. Wuhan and Changsha are the pilot cities for the policy of “resource-saving and environment-friendly society”, and have adhered to the model of resource conservation and green development, with a high level of living and well-being development. Therefore, they all show a high level of EWP. Medium urban hierarchy level cities are still in the stage of rapid economic development. Pursuing the goal of rapid economic development has led to a large amount of resources being input into the process and thus serious environmental pollution as a result. This extensive development model ignored social and environmental well-being, which led to a low level of EWP. Low urban hierarchy level cities, such as Tianmen, Xiantao, have less functions and play less roles. It is enough for them to satisfy their own development and therefore, its investment intensity, consumption level of resources, and environmental pollution is low, which has led to a high level of EWP. From the three-dimensional trend surface analysis results (Figure 3), the overall EWP of the study area showed a trend of higher levels in the southwest and lower levels in the northeast. Specifically, EWP showed a trend of higher levels in the west and lower in the east, as well as higher in the south and lower in the north. This trend was consistent with the EWP values of the three sub-urban agglomerations (Wuhan metropolitan area: 0.767; Changsha–Zhuzhou-Xiangtan urban agglomeration: 0.909; urban agglomeration around Poyang Lake: 0.671). The Changsha–Zhuzhou–Xiangtan urban agglomeration is located southwest of the study area, and its EWP was recorded at a top level. This resulted in the EWP level of the study area in the west and south directions recording higher than that in the east and north directions.

The central cities with high UH levels possessed a higher EWP, therefore showing a more coordinated “resource, environment, and human well-being” relationship. It has also produced a radiation-driving effect on the surrounding cities. However, the effect on more distant cities was not good. The following UH-tiered cities were still in the stage of rapid development. The relationship between its resources and environment and people’s well-being needs further improvement; these cities failed to play the leading role in their corresponding hierarchy level. Severe administrative barriers remained in the MRYRUA, making the EWP’s “collapse area” appear at the junction of sub-urban agglomerations or the border between provinces. Overall, the synergy of green development among cities in the study area was weak. A spatial polarization effect of EWP was observed, resulting in significant urban differences and spatial differentiation characteristics in the EWP. Strengthening the connection between middle and low hierarchy level cities and central cities in the region, improving development quality, enhancing people’s well-being, and improving the EWP, are all necessary.

### 3.2. Threshold Regression of Factors Influencing EWP

#### 3.2.1. Selection of Influencing Factor Variables

Cities at different UH levels have different natural and social environmental backgrounds. The correlation between EWP and its influencing factors is complex, and the probability of a non-linear relationship is high. To a certain extent, this phenomenon shows the possibility of the threshold effect in regard to the UH level. To explore the heterogeneity of the effects of various influencing factors, studies including those of Li et al. [30], Hu et al. [40], Wang et al. [34], and Fang and Xiao [32] were used for reference. Four aspects were selected as the influencing factors for the EWP of the MRYRUA.

The first aspect is technological progress. On the one hand, technological innovation helps to promote the organic integration of advanced technology and production processes. It can reduce resource waste and environmental damage in economic activities and increase economic output, ultimately improving EWP. On the other hand, new technologies generated by technological innovation can be divided into two categories: production technology and green technology. The former mainly affects production efficiency, while the latter mainly affects resource consumption and environmental pollution intensity. If more investment is used to develop production technology, it will expand the production scale. It will have a promotion effect on resource consumption and pollution discharge, thereby hindering the improvement of EWP. Therefore, technological progress is expected to have a bidirectional effect on EWP.

The second aspect is industrial structure, of which its upgrading reflects the replacement of production factors and the upgrading of production technology. These improvements have a meaningful impact on economic output and pollution emissions. The secondary industry is mainly dominated by industries with the most concentrated resource consumption and environmental pollution, such as the heavy chemical and construction industries. The more significant the proportion of the secondary industry, the more serious the pollution will be, and the lower the EWP. Therefore, the impact of the industrial structure is expected to be negative.

The third aspect is environmental regulation. According to the Porter hypothesis [80], reasonable government environmental regulation can stimulate enterprises to invest in technological transformation and environmental management innovation. The green transformation and upgrading of enterprises are forced, and the “innovation compensation” effect is generated so that the economic and environmental performance of enterprises can be improved simultaneously. Therefore, the expected impact of environmental regulation is positive.

The fourth aspect is population density. Population agglomeration can promote the efficient allocation and intensive utilization of resources, stimulate the formation of consumer markets, and promote urban economic development. When population agglomeration produces scale effects, marginal consumption and emission reduction costs can be reduced. Thus, a pollution reduction effect is formed [81], promoting the improvement of urban EWP. Further, the expected effect on population density is positive. In addition, the degree of openness, the government’s economic influence, and the intensity of urban construction were selected as control variables (Table 4). Owing to the lag of patent conversion, according to existing research [82], the lag period is two years. The patent authorization data in 2018 were selected as the input for technological progress. The threshold regression model was used to analyze each influencing factor’s hierarchical heterogeneity and staged effect on EWP.

The multicollinearity among the indicators was tested by the variance inflation factor (VIF) diagnostic method. As seen in Table 5, the VIF values of each indicator of the EWP-influencing factors were all less than five. That is, no multicollinearity occurred between indicators. These results mean that the selection of influencing factor indicators in this study is scientific, and the construction of the indicator system is reasonable.

#### 3.2.2. Analysis of Regression Results

The model took the UH level as the threshold variable, and the threshold test was performed on the core variables in turn (Table 6). Technological progress, industrial structure, environmental regulation, and population density passed the single-threshold test at the significance levels of 1%, 10%, 5%, and 5%, respectively. Environmental regulation passed the double-threshold test at the 5% significance level. Therefore, this study adopted the double-threshold test for environmental regulation and used the single-threshold test to analyze the remaining three core variables. The UH level threshold estimates and 95% confidence intervals for each core variable are shown in Table 7. The model parameter estimation results are shown in Table 8.

In model (1), the impact of scientific and technological progress on EWP presented a two-way and one-threshold feature. When the UH level was lower than 0.221, technological progress negatively, but not significantly, impacted the EWP. When the UH level exceeded the threshold, the elasticity coefficient of technological progress (0.0348) was positive and passed the 5% statistical level test. Five cities, including Wuhan and Changsha, were within the threshold. This result was as expected, confirming the bidirectional effect of technological progress on EWP. This means that for cities at different hierarchy levels, the effect of technological progress on EWP is different. High-level cities had a higher level of development and pursued green and high-quality development. The proportion of green technology investment in scientific and technological innovation was enormous, as such the positive effect of scientific and technological progress was more substantial, which is conducive to EWP. The scale of technology investment in low- and middle-level cities was relatively small; further, forming a scale effect was not easy. The aim of the investment was to undertake more technology transfer from high-level cities [83], as such it had a weak role in promoting EWP. The low-level cities were still pursuing economic development, and their investment in production technology was also relatively significant. This investment increased the economic benefits and was accompanied by the expansion of the production scale, thereby increasing pollution emissions and resource consumption. Therefore, these cities’ scientific and technological progress had no significant impact on EWP.

In model (2), the industrial structure represented by the proportion of secondary industry added value in GDP had a significant negative single-threshold characteristic on the impact of EWP. When the UH level was below 0.059, the elasticity coefficient of industrial structure on the EWP was −0.0466. Seven cities, including Pingxiang and Jingdezhen, were in this range. When the UH level exceeded the threshold, the elasticity coefficient of the industrial structure was −0.0530. Twenty-four cities, including Wuhan and Changsha, were in this range. This result was as expected. The higher the added value of the secondary industry, the more resource consumption and environmental pollution will be produced, harming the environment and EWP. The industrial structure of higher-level cities, therefore, had a more profound impact on EWP.

In model (3), the impact of environmental regulation on EWP presented a significant positive double-threshold feature. When the UH level was lower than 0.042, the elasticity coefficient of environmental regulation on EWP was 0.135, and five cities, including Yingtan and Xiantao, were below this threshold. When the UH level was between 0.042 and 0.237, the elasticity coefficient of environmental regulation was reduced to 0.129, and 22 cities, including Hengyang and Yichang, were in this range. When the UH level was higher than 0.237, the elasticity coefficient of environmental regulation was increased to 0.137. Four cities, including Wuhan and Changsha, crossed this threshold. The impact of environmental regulation on EWP presented a U-shaped characteristic with the change in UH level. For low hierarchy level cities, the cost and difficulty of environmental governance were relatively small, and the governance effect was more pronounced. As the UH level increases, the cost of environmental regulation and the difficulty of governance also increase, resulting in inefficient governance. Higher hierarchy level cities pursued the concept of coordinated development of “resources, environment, economy, and society,” and their environmental regulations were stringent. Furthermore, they were supported by a profound economic and technological foundation, enhancing the effect of environmental regulation and making it more prominent.

In model (4), the effect of population density on EWP presented a significant positive single-threshold characteristic. When the hierarchy level was below 0.221, the elasticity coefficient of population density on EWP was 0.445, and 26 cities, such as Yichang and Shangrao, were within this range. After crossing this threshold, the elasticity coefficient of population density on EWP rose to 0.930, and five cities, including Wuhan and Changsha, were within this range. Higher hierarchy level cities had the scale effect and agglomeration effect of population agglomeration. A higher population density was conducive to promoting the intensive use of resources and improving economic benefits and EWP. However, for lower hierarchy level cities, scale and level limited the scale effect of population agglomeration and comprehensive effect. Thus, the impact of population density on EWP was weaker than in high hierarchy level cities.

Among the four models, most of the elasticity coefficients of the degree of openness, government economic influence, and urban construction density on EWP did not pass the significance level test. The elasticity coefficients of the degree of openness in models 1, 3, and 4 were all negative, showing an insignificant inhibitory effect. The reason for this is that China restricted the transfer of highly polluting resource-intensive industries to its domestic area. Some international capital has also turned its attention to Southeast Asia rather than China. This means that the MRYRUA was no longer a “pollution sanctuary” for international capital. Furthermore, China is currently actively building a large domestic market cycle, meaning that the influence of foreign capital will be reduced further. Therefore, openness failed to have a significant impact on EWP. The elasticity coefficients of government financial influence in models 1, 3, and 4 were all positive, but the elasticity coefficient was not significant. The construction of an ecological civilization is an essential indicator for evaluating the functions of local governments in China. Governments with stronger financial influence can invest more labor and funds in public utilities such as environmental protection, education, and medical care. It can promote environmental friendliness, enhance public welfare and people’s well-being, and improve EWP. The elasticity coefficient of urban construction intensity in models 3 and 4 was positive, and the elasticity coefficient in models 1 and 2 was negative. Nevertheless, the coefficient only passed the 10% significance level test in Model 4. The higher the urban land use intensity, the more effective the land use can be. However, at the same time, there will also be inefficient sprawling developments, resulting in a low degree of intensive utilization and various ecological and environmental problems. Under these two effects, the urban construction intensity of the MRYRUA failed to impact EWP significantly.

## 4. Discussion

Sustainable development is one of the most critical issues in the world. The SDGs cover natural resources, the environment, human well-being, and other aspects. It shows that EWP is an effective tool to measure the progress of the SDGs. The expansion of cities, the growth of industries, and the increase in population have brought many pressures on local resources and the ecological environment, which have greatly affected the region’s sustainable development. An urban agglomeration or megalopolis is a highly integrated cluster of spatially compact and economically highly integrated cities. The high degree of integration of cities makes urban agglomerations one of the world’s most important carriers of human production, life, and economic development [13]. Paying attention to the improvement of EWP is crucial to the sustainable development of urban agglomerations. The urban agglomerations and megalopolises in developed countries, such as the Great Lakes Megalopolis in the United States, the London urban agglomeration in the United Kingdom, and the Pacific Rim urban agglomeration in Japan, have entered a mature stage of development. They face relatively little pressure on the population, resources, and environment [52]. However, most urban agglomerations or metropolitan areas in developing countries such as China are in the embryonic stage and therefore the rapid growth stage [84]. They face tremendous pressure in regard to population, resources, and the environment and, correspondingly, have many more development problems. Further, the development and rapid expansion of urban agglomerations have brought enormous pressure on the ecosystem [85]. Therefore, solving the uncoordinated economic, social, and ecological development is urgently needed in order to promote sustainable development. Promoting EWP can promote the sustainable development of urban agglomerations. However, in the existing studies, there are no studies that analyze the sustainability of rapidly developing urban agglomerations through EWP. Compared with the existing studies, this study explored the spatial differentiation law of EWP in the MRYRUA from a hierarchical perspective. This study also empirically tested the heterogeneity of EWP-influencing factors on the direction and size of EWP in different UH levels.

Significant differences were found in EWP levels among the cities within the MRYRUA. First, the UH level was not entirely consistent with the EWP level for most cities. Some cities at the middle and high UH levels had lower levels of EWP, while others at the lower levels had higher levels of EWP. This finding is similar to that of Knight et al. [25] who found differences in EWP between developed and less developed countries. This similarity shows that our study’s indicators and methods of EWP measurement are reasonable. Knight et al. [25], Dietz et al. [28], and Long et al. [16] studied only the relationship between economic development and the EWP. This study reveals the relationship between urban hierarchy and EWP from the city system. Second, the EWP of the MRYRUA presented a multi-center “core–edge” spatial distribution. Xia and Li [35] found that the EWP of the Beijing–Tianjin–Hebei urban agglomeration also had a similar spatial differentiation pattern. By comparison, this study further discussed the UH level, enriching the research perspective of the EWP in urban agglomerations. Third, threshold regression results showed a non-single linear relationship between the influencing factors and EWP in the MRYRUA. The influencing factors also showed different functional characteristics in different urban tiers. Deng et al. [37] found that the influencing factors of EWP in China’s provinces had different effects on EWP in different regions, which are consistent with the results of our study. However, Deng et al. only subjectively, and simply, divided the study area into three regions: east, middle, and west. In our study, the UH was used as the threshold variable to divide the threshold interval objectively. As such, the results of our study are more precise and scientific. Technological progress had a positive effect on the EWP of high UH level cities, but had an insignificant, though negative, effect on the EWP of middle and low UH level cities. This result is not found in existing research. The effects of industrial structure, environmental regulation, and population density were consistent with most existing studies [16,30,40,86]. However, their studies only analyzed the general situation of all regions and did not analyze the specific differences between regions. By contrast, our study identified the stage characteristics of each influencing factor at different UH levels from the perspective of hierarchy. It provides a new perspective to explain the phenomenon of differences in EWP in different cities.

From the hierarchy perspective, large-scale megalopolises, urban agglomerations, or small-scale metropolitan areas all form a hierarchical system of cities on different scales. Cities with different UH levels have different scales and positions in the UH system and also have different functions and roles. On this basis, a hierarchical effect is formed, which acts on the process of transforming ecological resources and the environment into an increase in human well-being. On the one hand, the highest level city in terms of hierarchy in the urban agglomeration play a critical leading role in the region. Higher ecological welfare performance means a coordinated “economic–social–environmental” development model. This model has a guiding role for cities at other levels and has diffusion and spillover effects on other cities as well. The sub-core or satellite city at the next hierarchy level assumes the medium function and expands the spatial scope of the diffusion effect of the high hierarchy level city. At the same time, they also need to improve their own EWP. On the other hand, affected by the hierarchical effect, the effects of various influencing factors differ between high-hierarchy and low-hierarchy cities. For example, the impetus of technological progress to EWP is more significant in high-hierarchy cities but weaker in low-hierarchy cities. Environmental regulation presents U-shaped among high, middle, and low hierarchy cities. Being alone in improving EWP is difficult for cities in urban agglomeration areas. No one-size-fits-all, immutable path exists for improving EWP. The role of cities at all hierarchy levels needs to be considered. Implementing local policies and strengthening collaboration to improve the relationship between the economy, society, and the environment is necessary.

Exploring the spatial differentiation law of the EWP of the MRYRUA and the heterogeneity of influencing factors from a hierarchical perspective is of great theoretical and practical significance. This research provides a new perspective on promoting the sustainable development of urban agglomerations. This study is beneficial to the rapidly developing urban agglomerations in China, and other developing countries, in playing an overall better role in the urban agglomeration hierarchy system. This study can also better solve the problem of incongruity between economic development, resource, environment, and human well-being improvement. The government can propose targeted and differentiated EWP improvement paths in the future according to different UH levels.

This study still has some limitations in expressing residents’ true well-being. Owing to the limitation of data acquisition, this study did not consider subjective well-being indicators such as resident satisfaction when measuring human well-being. The number of core explanatory variables in regression analysis is limited, and more influencing factors can be added for more in-depth research in the future. The EWP research framework constructed in this study reflects the relationship between resource and environmental consumption, economic growth, and well-being improvement. However, it did not define the constraints of ecological boundaries. High EWP levels that break ecological boundaries, or cities with a shallow level of well-being are both unsustainable. A low level of well-being undermines the ideal level of human development. Excessive ecological input will cause severe damage to the ecological environment that is difficult to reverse. In the future, the upper safety limit of ecological consumption and the just lower limit of human well-being development guarantee should be considered. EWP must be explored within the safe and just spatial boundaries of human social development.

To improve the EWP of the MRYRUA, to promote the construction of ecological civilization, and the high-quality development of the urban agglomeration, the following suggestions are put forward.

(1) The investment in scientific and technological innovation should be strengthened, and the allocation of innovation resources should be optimized. The capacity building of scientific and technological absorption as well as re-innovation should be expanded, and the transformation rate of scientific and technological achievements should be improved. According to the hierarchy level, different scientific and technological innovation strategies should be formulated to promote the flow of scientific and technological innovation elements and regional innovation cooperation. Collaborative innovation policies should be developed between neighboring cities. The innovation chain between cities should be opened up and industry–university–research cooperation between cities should be strengthened.
(2) The vital role of industrial structure optimization in promoting technological innovation and industrial upgrading should be given full play. The development of the industrial structure should be promoted in the direction of rationalization and advancement. The development of green industries should also be accelerated. The important role of green and low-carbon industries in promoting regional development and managing the ecological environment should be enhanced.
(3) Environmental pollution remains the bottleneck restricting the improvement of EWP. Cities at all levels need to strengthen environmental regulation further. Considering EWP’s “efficiency” and “fairness” in the future is necessary. A multi-party collaborative governance model for the ecological environment should be built. In the pollution control process, the government, enterprises, the public, and other social parties should be guided to participate together.
(4) The restrictions on the urban population size should be properly lifted to give full play to the scale effect and intensive effect of the population. Intensive use of urban land and sprawling development should also be avoided.
(5) The government of middle and low hierarchy level cities should be encouraged to transition from pursuing the traditional GDP target to the goal of improving people’s comprehensive well-being. The public’s well-being in income, health care, education, and ecological environment should be improved. The problem of imbalance and insufficiency should be solved, people’s growing needs for a better life should be met, and people’s life satisfaction should be improved.

## 5. Conclusions

Based on the Super-SBM model, this study measured the urban-scale EWP of the MRYRUA in 2020 and analyzed its spatial differentiation characteristics. The threshold regression model was used to empirically test the heterogeneity and stage of the influencing factors of EWP at different UH levels. The main conclusions obtained are as follows.

(1) The EWP of the MRYRUA showed significant spatial differentiation, and the overall trend was highest in the southwest and lowest in the northeast. The “core–periphery” situation with Wuhan, Changsha, and Nanchang as the core was apparent, and the phenomenon of “central collapse” occurred at the junction of sub-urban agglomerations. The hierarchy level of the city was not consistent with the EWP level. The high hierarchy level central cities of the urban agglomeration had higher EWP levels. Significant differences in performance levels were found between cities. The overall spatial differentiation characteristics indicate that the MRYRUA had a soft green and coordinated development capability; as such, spatial polarization of the EWP appeared. Promoting interactive and coordinated development among the cities in the MRYRUA is urgently needed. Strengthening the diffusion and radiation-driving effect of high-hierarchy-level cities is necessary.
(2) A non-single linear relationship was found between the influencing factors of EWP and EWP in the MRYRUA. The impact of technological progress, industrial structure, environmental regulation, and population density on the EWP of the MRYRUA all showed threshold characteristics. In different UH intervals, influencing factors have different effects on EWP.
(3) For different UH levels, the influencing factors of the EWP are heterogeneous and staged. The impact of technological progress, industrial structure, and population density showed a single threshold characteristic. The impact of environmental regulation showed the characteristics of double thresholds. Technological progress presented two-way, single-threshold, and two-stage characteristics. The industrial structure presented a significant negative single-threshold dual-stage feature. Environmental regulation presented a very significant positive double-threshold three-stage feature. Population density exhibited a significant positive single-threshold two-stage feature.

## Figures and Tables

**Table 1:** Input–output evaluation index system of ecological well-being performance (EWP).

Dimension	Criteria	Indicator	Indicator Interpretation	Literature Support
Natural resource consumption	Land consumption	Per capita built-up area (km^2^/person)	Reflects the input level of land resources	Wang et al. [18]; Xia and Li [35]
	Water consumption	Per capita water consumption (m^3^/person)	Reflects the level of water resources input	Wang et al. [18]; Yao et al. [56]
	Energy consumption	Per capita electricity consumption (kW·h/person)	Reflects the level of energy consumption	Bian et al. [26]; Xia and Li [35]
Ecological environment destruction	Wastewater	Per capita industrial wastewater (t/person)	Reflects the degree of water pollution	Yao et al. [56]
	Waste gas	Per capita nitrogen oxide emissions (t/person)	Reflects the degree of air pollution	Bian et al. [26]
	Solid waste	Per capita industrial solid waste generation (t/person)	Reflects the pollution degree of solid waste	Wang et al. [18]; Xia and Li [35]
Human well-being output	Economic growth	Per capita GDP (CNY)	Reflects the well-being of material wealth	Long et al. [16]; Li et al. [30]
	Social equality	Per capita disposable income of rural residents/per capita disposable income of urban residents	Reflects the well-being of social equity	Lu and Wang [58]
	Universal education	Average years of education (year)	Reflects the well-being in education	Li et al. [30]; Fang and Xiao [32]
	Health care	Number of beds in health institutions per 10,000 persons (bed/10,000 persons)	Reflects medical and health well-being	Xia and Li [35]; Hu et al. [40]
	Favorable environment	Per capita green park space (m^2^/person) Excellent air quality rate (%)	Reflects the well-being of a beautiful environment	Li et al. [30]; Hu et al. [40]

**Table 2:** Evaluation index system of urban hierarchy level (UH).

Dimension	Criteria	Indicator	Indicator Interpretation	Weight	Literature Support
Size and scale level	Population scale	Urban population (10,000 person)	Reflects the population size of the city	0.051	Yao et al. [63]
	Economy of scale	GDP (billion CNY)	Reflects the economic scale of the city	0.088	Yao et al. [63]
	Spatial scale	Urban construction land area (km^2^)	Reflects the spatial scale of the city	0.111	Yao et al. [63]
Impact and role level	Transportation	Road freight (10,000 t)	Reflects the traffic function of the city	0.056	Fan et al. [73]; Commendatore et al. [61]
		Total road mileage (km)	Reflects the traffic function of the city	0.047	Fan et al. [73]; Commendatore et al. [61]
	Education function	Number of undergraduate students (person)	Reflects the educational role of the city	0.157	Wang et al. [71]
		Number of full-time teachers in colleges and universities (person)	Reflects the educational role of the city	0.167	Wang et al. [71]
	Science and technology function	Number of patents granted (pcs)	Reflects the role of science and technology of the city	0.116	Wang et al. [71]
	Business service	Retail sales of social consumer goods (billion CNY)	Reflects the commercial role of the city	0.088	Zhou et al. [60]
	Social service	Number of health technicians (person)	Reflects the public service function of the city	0.065	Zhou et al. [60]
		Number of hospital beds (bed)	Reflects the public service function of the city	0.054	Zhou et al. [60]; Yao et al. [63]

**Table 3:** The EWPs and UHs of urban agglomeration in the middle reaches of the Yangtze River (MRYRUA) in 2020.

City	EWP	UH	City	EWP	UH
Changsha	1.591	0.687	Yingtan	0.551	0.022
Tianmen	1.538	0.007	Jingzhou	0.543	0.163
Wuhan	1.260	0.967	Xianning	0.497	0.085
Xiantao	1.236	0.015	Yueyang	0.485	0.165
Pingxiang	1.217	0.044	Xiangtan	0.427	0.111
Ezhou	1.133	0.012	Jingdezhen	0.422	0.042
Shangrao	1.079	0.191	Jian	0.417	0.140
Loudi	1.046	0.093	Xiaogan	0.406	0.111
Hengyang	1.044	0.221	Yichang	0.383	0.195
Yìyang	1.016	0.117	Jingmen	0.375	0.075
Huanggang	1.011	0.163	Xinyu	0.371	0.059
Fuzhou	1.009	0.114	Huangshi	0.361	0.080
Changde	1.006	0.180	Jiujiang	0.334	0.188
Nanchang	1.006	0.433	Yichun	0.306	0.174
Zhuzhou	0.661	0.158	Changsha-Zhuzhou-Xiangtan urban agglomeration	0.909	0.216
Xiangyang	0.645	0.237	Wuhan metropolitan area	0.767	0.163
Qianjiang	0.579	0.008	The urban agglomeration around Poyang Lake	0.671	0.141

**Table 4:** Influencing factors of EWP in MRYRUA.

Influencing Factors	Variable Category	Indicators	Symbol
Technological progress	Core variables	Number of patent authorizations per 10,000 people (pieces/10,000 people)	TP
Industrial structure	Core variables	The proportion of secondary industry in GDP (%)	IS
Environmental regulation	Core variables	Centralized sewage treatment rate (%)	ER
Population density	Core variables	Population density in built-up area (10,000 people/square kilometer)	PD
Degree of openness	Control variable	The proportion of foreign capital utilized in GDP (%)	FDI
Government economic influence	Control variable	Local fiscal expenditure as a percentage of GDP (%)	GE
Urban construction intensity	Control variable	The proportion of urban built-up area in urban area (%)	UDI

**Table 5:** Variance inflation factor independence test.

Variable	VIF	1/VIF
FDI	2.77	0.361
TP	1.94	0.516
UDI	1.79	0.560
GE	1.75	0.570
PD	1.68	0.596
IS	1.41	0.709
ER	1.23	0.810
Mean	1.80	

**Table 6:** Test on threshold effects.

Threshold Inspection	TP	IS	ER	PD
Single threshold test	7.359 **	3.888 *	6.482 **	7.193 **
	(0.033)	(0.070)	(0.037)	(0.017)
Double threshold check	3.164	3.373	10.568 **	0.521
	(0.170)	(0.157)	(0.013)	(0.620)
Triple threshold test	1.951	0.713	0.293	2.887
	(0.253)	(0.520)	(0.627)	(0.187)

Note: The numbers above the brackets are the F statistics corresponding to the threshold test; **, and * indicate the significance levels of 5%, 10%, respectively; the numbers in the brackets are the *p* values. These values were obtained by the bootstrap method, and the number of bootstraps used was 300.

**Table 7:** Threshold estimates.

Threshold Estimate	TP	IS	ER	PD
The first threshold estimates δ1	0.221	0.059	0.042	0.221
	[0.042, 0.221]	[0.042, 0.221]	[0.042, 0.075]	[0.085, 0.221]
The second threshold estimate δ2			0.237	
			[0.237, 0.237]	

Note: The numbers above the square brackets are the threshold estimates; the numbers in the square brackets are the 95% confidence intervals.

**Table 8:** Regression results of threshold model.

	Model 1	Model 2	Model 3	Model 4
Independent variable	TP	IS	ER	PD
Threshold variable	UH	UH	UH	UH
FDI	−0.0627	0.0575	−0.105 **	−0.0439
	(−1.24)	(1.32)	(−2.31)	(−1.06)
GE	0.00299	−0.0504	0.0523	0.015
	(0.10)	(−1.66)	(1.56)	(0.52)
UDI	−0.00374	−0.00316	0.00866	0.0183 *
	(−0.42)	(−0.35)	(1.00)	(1.93)
T(UH ≤ δ1)	−0.0128	−0.0466 ***	0.135 ***	0.445 ***
	(−0.74)	(−3.11)	(3.11)	(3.67)
T(UH ≥ δ1) or (δ1 ≤ UH ≤ δ2)	0.0348 **	−0.0530 ***	0.129 ***	0.930 ***
	(2.10)	(−3.34)	(3.05)	(3.71)
T(UH > δ2)			0.137 ***	
			(3.18)	
Constant	0.970 ***	3.291 ***	−12.28 ***	−0.00893
	(3.25)	(4.33)	(−2.89)	(−0.02)
R2	0.301	0.340	0.446	0.425
F	2.245	2.576	3.214	3.697

Note: The numbers above the parentheses are the coefficient values; ***, **, and * indicate the significance levels of 1%, 5%, and 10%, respectively; the numbers in the parentheses are the t values.

## Data Availability

Not applicable.
